# Developing and preliminary testing of temporal occlusion decision scenarios in dynamic contexts: a three-step method

**DOI:** 10.3389/fpsyg.2026.1893159

**Published:** 2026-08-11

**Authors:** Brian McGuigan, Linus Andersson, Stefan Holmström, Annika Johansson, Erik Lundkvist

**Affiliations:** 1 Department of Psychology, Umeå University, Umeå, Sweden; 2 Unit for Police Work, Umeå University, Umeå, Sweden

**Keywords:** decision making, expertise, police decision making, police research, scenario based method, temporal occlusion

## Abstract

Temporal occlusion is a method to segment recorded or created decision scenarios into constituent parts, to enable studying judgment and decision-making. There is increasing interest in using the method in complex domains such as police education, which calls for guiding principles in constructing and evaluating scenarios. We suggest principles for a three-step method to produce temporally occluded scenarios, comprising (1) *extraction*: how to identify and systematically select relevant scenarios, (2) *production*: how to develop materials, and (3) *testing*: iterative pilot testing of the materials. Further, we exemplify the method by producing and testing scenarios relevant for police work. In the *extraction* step, potential scenario descriptions and relevant research questions and outcome variables were decided upon after a discussion with domain experts. After evaluation, a subset of scenarios was *produced* as videos and temporally occluded, to allow for stopping scenarios at key points and asking for predictive judgments and plausible courses of action. In the *testing* step, materials were iteratively pilot tested, resulting in the finalized set of temporally occluded materials. This three-step process and the preliminary results provide initial guidance contributing to the standardization of temporally occluded scenario development that can help researchers gain a better understanding of anticipation and decision-making in different domains. Applications for this three-step method and future research possibilities are discussed.

## 1 Introduction

A key discrepancy between laboratory-based and real-world judgment and decision-making research regards methodological constraints. While the laboratory allows a high degree of experimental control, with tasks that can be formulated as normatively correct or incorrect, real-world problems tend to be dynamic, ill-defined, and time pressured. They are often studied retrospectively through different forms of self-reports, or through vignette scenarios. Yet, critical aspects of everyday decisions may be identified and formalized, which opens venues to create decision scenarios that are both controlled and true to life (Aguinis and Bradley, 2014).

Temporal occlusion is a widely used method for segmenting complex recorded or created scenarios into constituent parts. At key time points, the scenario stops, and the viewer is asked to predict upcoming events or outcomes (Müller et al., 2015). This procedure has been used to study predictive judgments across different skill levels, particularly in dynamic contexts like sports, driving, and aviation (Kujala et al., 2023; Suss and Ward, 2015). For example, skilled tennis players have been shown to perform better than novices at predicting serve direction when visual information is limited (Farrow et al., 2005). Similar patterns of superior expert prediction have been observed in a range of sports using temporal occlusion tasks (Song et al., 2025).

In the law enforcement domain, temporally occluded videos of staged police–citizen encounters have shown that skilled officers (i.e., officers with substantial experience and training) are superior at decision-making compared to novices (Suss and Ward, 2012, 2018). Furthermore, research using real video footage of police incidents (i.e., recordings from body-worn cameras) that were temporally occluded has been used to study differences in how more and less experienced officers assessed appropriate use of force (Mangels et al., 2020; Ta-Johnson et al., 2025). Outcomes of both studies revealed that more experienced officers were less inclined to use force but also highlighted the feasibility of using temporal occlusion as a method for studying judgments, and for the purpose of training.

Hence, temporal occlusion has also been successfully used in scenarios with greater complexity than, for example, predicting ball trajectories. The added complexity is, however, associated with a need for methodological stringency; a sentiment in line with arguments from Ta-Johnson et al. (2025) for method development in terms of, e.g., scenario representativeness and scope. Currently, there is a lack of a systematic process for producing and testing temporally occluded scenarios for complex situations that can be adapted to different domains. Establishing such a systematic, structured process could provide valuable knowledge about skilled performance that can be applied to training, safety measures, and standard procedures in a range of domains.

### 1.1 A three-step method to produce temporally occluded materials

Best practice recommendations on vignette or thought experiment studies often describe a stepwise, iterative procedure from inception, to implementation, to discussing implications of the results (Aguinis et al., 2023; Aguinis and Bradley, 2014). We expand on this general principle and argue for a three-step method when developing temporally occluded scenarios, comprising (1) extraction, (2) production, and (3) testing.

#### 1.1.1 Extraction

Arguably the first, necessary, step to formalize everyday judgments is to identify and extract their constituent parts. While this may be obvious in some situations, like when someone is hitting a ball, key features of more complex situations may be difficult to discern. Moreover, it is of importance that the chosen example scenario represents the problem or judgment to be studied. Klein (2015) argues that the best real-world scenarios are constructed by experts who have tacit domain knowledge, and who often make judgments based on intuition. While the boundaries of these constructs are difficult to pin down (Gourlay, 2006) and partly overlap (Smith, 2001), tacit knowledge and intuition allude to practical “know-how” based on extended practice and personal experience that can be, but often is not, explicit in nature. This means that expert knowledge needs to be extracted through other means than just asking.

Cognitive Task Analysis is a suitable methodology in this regard. It is commonly used to uncover and understand mental models underlying tacit knowledge and skilled performance in complex tasks (Crandall et al., 2006). In short, Cognitive Task Analysis is a framework that involves early engagement with experts to identify pertinent conflict or judgment situations, and to formalize their constituent phases, such as key turning points. Further, experts are asked to reason about key goals, skills, and other factors that may critically influence outcomes. Cognitive Task Analysis has, for instance, been used to inform scenario development in complex, high-stakes situations such as military-civilian encounters (Klein and Borders, 2016). Cognitive Task Analysis interviews helped identify incidents that informed scenario construction, after which a panel of subject matter experts worked through the scenarios, ranked the response options, and provided rationales (Klein and Borders, 2016; Klein et al., 2015). Based on these arguments and results, we suggest keeping the following in mind when extracting scenarios to be temporally occluded:

*1: Involve domain experts at a very early stage—in formalizing relevant research questions, and key outcome* var*iables.*

*2: Discuss a wide range of possible scenarios with the experts and choose which ones to implement based on their judgments of key features such as relevance, representativeness, and the potential to differentiate skill levels in accordance with CTA principles.*

#### 1.1.2 Production

Several forms of media can be used to produce temporally occluded scenarios, including recorded or AI produced video, virtual reality, and audio. While these choices afford different levels of immersion—a factor regarded as important for participant engagement (Aguinis and Bradley, 2014)—the choice is ultimately constrained by practical issues. Although scenarios may be complex, key events or turning points may be (and should be) few, and temporal occlusion points should be designed to precede these events. In complex domains such as law enforcement, a critical aspect that needs to be considered when creating video stimuli is whether it is representative of real tasks that are encountered by the practitioners being studied (Suss and Boulton, 2019). In addition, video stimuli realism can be enhanced by using experts (e.g., police officers) with experience as actors or to offer instructions to actors because they are knowledgeable about common behaviors displayed by suspects and dealing with weapons (Suss and Boulton, 2019). Suggestions to produce temporally occluded scenarios are:

*3: Base the decision on what scenarios to be selected on both expert judgments and practical constraints.*

*4: Domain experts should be involved also in the creation of the temporal occlusion scenarios, by taking part in,* e.g.*, recordings or material production.*

#### 1.1.3 Testing

The third step comprises testing the produced scenarios, to probe the feasibility of both materials and outcomes. While the forms of testing should be based on the requirements of the research question or study, important principles may be extracted from the literature on usability testing (Salvendy and Karwowski, 2021). These include rationales for recruiting usability test participants, deciding what output to collect, as well as the benefits of iterative usability testing. Klein et al. (2013) conducted pilot testing with a representative group by iteratively improving feasibility and altering the methodology after each pilot testing. This iterative pilot testing enabled Klein and Borders (2016) to identify procedural misunderstandings and inform design decisions so that they could formalize the procedures in their methodological approach, which they referred to as ShadowBox Training. In addition, Suss and Boulton (2019) emphasize the importance of pilot testing procedures that will be used with expert police personnel because highly experienced officers or experts can be difficult to recruit due to their limited numbers and busy schedules. Suggestions when testing the feasibility of materials and procedures are to:

*5: Decide how to conduct the usability testing based on either previous research with domain experts or in close collaboration with domain experts, by selecting relevant outcome measures, and methods to collect data.*

*6: Recruit and test participants that are representative of the population to be studied.*

### 1.2 Current study

The current study has a twofold aim. First, we present and motivate a structured three-step method—comprising extraction, production, and testing—for developing temporally occluded decision scenarios that can be adapted across domains. Second, we illustrate the method by applying it in a police patrol context, where each step is implemented and reported as a separate part of the paper.

Police patrol work was chosen as the illustrative domain because it exemplifies the type of dynamic, ill-defined, and time-pressured situations that temporal occlusion is well suited to study (Suss and Ward, 2018), and because there is a small but growing body of work using temporal occlusion in law enforcement to draw on (Mangels et al., 2020; Suss and Ward, 2012, 2018; Ta-Johnson et al., 2025). At the same time, the principles and procedures we outline are intended to be transferable to other domains characterized by similar decision-making demands, such as emergency medicine, firefighting, or military operations. The transferability of the three-step process to other domains is conceptual in the current study and has not been empirically tested. Likewise, empirically validating the resulting instrument, including any test of expert/novice differentiation, is beyond the scope of the present methods paper.

The paper is organized around the three steps of the method. In Part 1 (Extraction), a workshop with expert police teachers was used to identify candidate scenarios and to specify the variables on which scenarios would later be evaluated. In Part 2 (Production), a larger group of expert police teachers evaluated the candidate scenarios in a survey, after which a subset was selected based on these ratings together with practical considerations, and the selected scenarios were then scripted, recorded, and temporally occluded. In Part 3 (Testing), the resulting materials and the measure were pilot tested with police students and police teachers to evaluate feasibility, clarity, and time demands. Each part is presented with its own brief Method and Results section, and the implications of the three-step method as a whole are addressed in the General Discussion.

### 1.3 Ethics statement

The study was approved by the Swedish Ethics authority (approval number: 2023-01509-01; decision date: 2023-05-02).

All participants were informed about the purpose of the study, the procedures involved, and their rights as participants prior to participation.

Actors and police students participating in the filming of the video scenarios provided written informed consent, including consent for being recorded and for the use of the video material in research and training contexts.

Police students participating in the pilot testing were provided with information about the study before participation and were debriefed afterward. Participation was voluntary, and informed consent was obtained prior to data collection.

Video material was handled in accordance with institutional and ethical guidelines. Recordings were securely stored on password-protected systems accessible only to the research team and were used solely for research purposes in line with the approved study.

Participation involved minimal risk. The scenarios were staged and acted, participation was voluntary, and participants could withdraw at any time without consequence.

## 2 Part 1: extraction

### 2.1 Method

#### 2.1.1 Participation

Expert police teachers (*n* = 4) were recruited from the Unit for Police Work at Umeå University. The experts were recruited using criterion-based convenience sampling, and inclusion criteria required participants to be active or former police officers with experience in Swedish police patrol work. They had an average of 14 years of experience in operational police work (range = 10–20 years), with some also having experience in regional task forces, and an average of 13 years of experience as police teachers (range = 8–25 years). All experts had extensive experience teaching core police subjects such as firearms, tactics, and self-defense, and were certified Polkon instructors or supervisors.

#### 2.1.2 Procedure

The expert police teachers were invited to a workshop session to (1) identify and discuss potential scenarios to be produced in Part 2, and (2) decide variables of interest to base scenario selection on. We based the scenario selection discussion in part on previously used scenarios (Brown and Daus, 2015; Dahlman et al., 2016; Flin et al., 2007) but also encouraged the formulation of novel scenarios. The experts were further asked to suggest variables of relevance for scenario selection. Key takeaways from the discussion were summarized and shared with the experts to ensure that they corresponded to their views. Hence, the procedure was inspired by suggestions 1 and 2.

#### 2.1.3 Results

The experts pointed out that patrol police often face situations characterized by limited information and time constraints. Therefore, they suggested that the scenarios to be developed should fit this description. The expert police teachers further argued that video scenarios were a suitable medium for police encounters because of the many details and factors that need to be taken into consideration.

##### 2.1.3.1 Potential scenarios

Based on the previous materials and the experts’ novel suggestions, a list of ten potential, summarized scenario descriptions were compiled, all culminating in violent escalations. See Supplementary material S1 for descriptions of all 10 potential scenarios.

##### 2.1.3.2 Variables for scenario selection

It was decided that the outcome variables of interest with the scenario selection would be (1) *realism*, i.e., how realistic and representative they are regarding Swedish patrol police work; and (2) *skill differentiation*, i.e., whether scenario outcomes could be used to rank or judge police decision-making skill levels.

## 3 Part 2: Production

### 3.1 Method

#### 3.1.1 Participants

Expert police teachers (*n* = 9; M age = 50.4) with an average of 25 years of police work experience (range = 13–37 years) were recruited from different parts of Sweden. The experts were recruited through criterion-based convenience sampling, and inclusion criteria were that participants had to be active or former police officers with experience in Swedish police patrol work. The expert police teachers had professional experience across multiple domains of police work, including frontline patrol, investigations, command roles, and specialized units (e.g., tactical operations, narcotics, and intelligence), as well as police training and education. Many had also held leadership positions and worked across multiple regions of Sweden.

#### 3.1.2 Materials

##### 3.1.2.1 Scenario selection based on realism and skill differentiation

**Figure 1:**
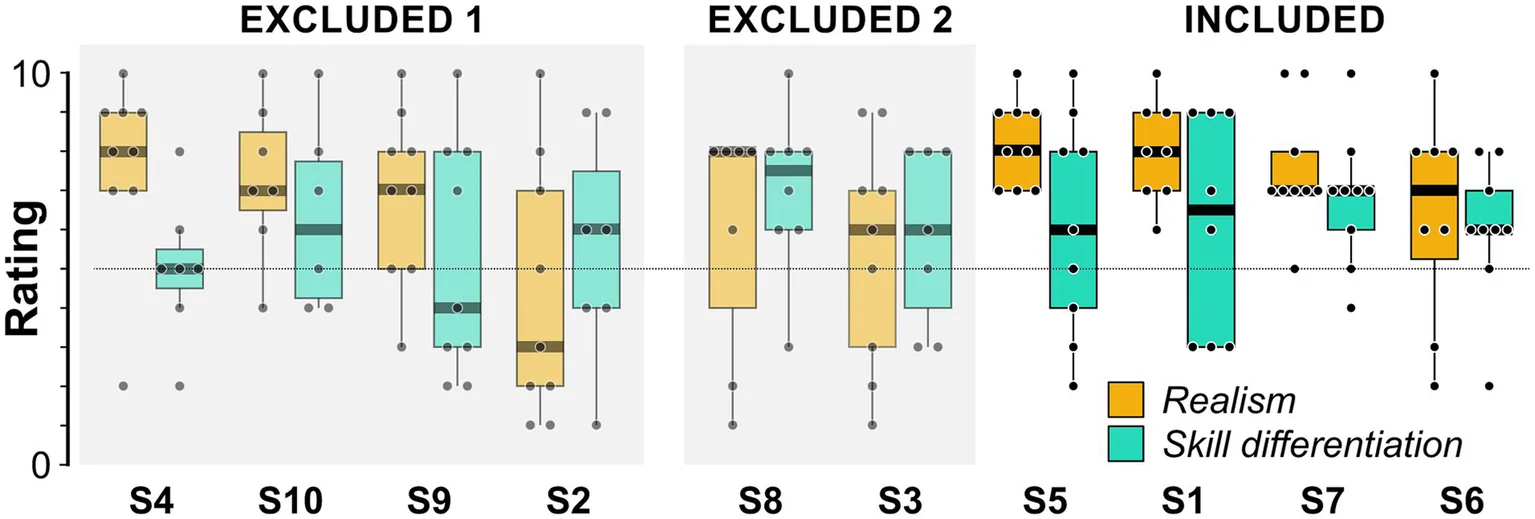
Scenario ratings (realism in orange; skill differentiation in green). Boxes represent interquartile range (25th–75th percentiles), whiskers represent the most extreme values within 1.5 × IQR, and short horizontal bars indicate the median. Individual ratings are overlaid as points. “Excluded 1” indicates scenarios excluded during Production (Part 2). “Excluded 2” indicates those excluded during Testing (Part 3). “Included” indicates the four video scenarios retained in the final measure.

We created a survey consisting of the 10 scenario descriptions from Part 1. For each scenario, participants rated its *realism* and potential for *skill differentiation* from 1 to 10, where 1 was “*poor*” and 10 was “*excellent*.” The expert ratings were summarized descriptively using boxplots, and the dispersion in the ratings is illustrated in Figure 1. Inter-rater agreement indices were not calculated because the ratings were used to inform scenario selection rather than to assess measurement reliability. Following each scenario, participants had the opportunity to answer a free-text question on how to improve both aspects. The survey further included questions on age, years of experience, and a free-text question about their police background. Microsoft Forms was used as a data collection platform. Scenarios were selected based on (1) rated *realism*, discarding scenarios below a mean of 5 to ensure that they were at least moderately realistic; (2) rated *skill differentiation*; and (3) variation, to illustrate a range of situations, and (4) practical constraints.

The decision rules used for scenario selection in the current study began with screening scenarios that had realism ratings below a mean of 5, because we wanted to ensure that each selected scenario was deemed at least moderately realistic and faithful to situations encountered in the field. After the initial realism screening, potential skill differentiation was given greater weight in the selection process, as scenarios with higher rated potential to differentiate were expected to offer more variation in courses of action, and therefore provide more information for studying decision-making. In line with the suggested principles, practical constraints were operationalized as factors such as participant understanding of the scenario descriptions, feedback from open-ended responses, and the requirement that scenarios include at least two substantive comments on skill differentiation to support their refinement. These factors, together with variation, were considered alongside scenarios with the highest ratings for skill differentiation during the selection process. For more information about this process in the police context (e.g., survey rating items and more detailed decision rules), see Supplementary material S1.

##### 3.1.2.2 Recording equipment and resources

We decided to use recorded video as a medium for scenario creation based on recommendations from the workshop discussion in Part 1, and previous research. Scenarios were filmed from a first-person point of view with a head-mounted Go Pro camera. Six paid actors were hired to portray suspects, victims, and bystanders, and six police students participated voluntarily to portray the police officers in the video scenarios. More information about script development, script examples, filming procedures, technical information, exact timing of occlusion points and culminating events for each scenario, and video editing can be found in Supplementary material S2. As the content of the recorded scenarios were based partly on the survey outcomes, additional information on material production is described under “Procedure.”

#### 3.1.3 Procedure

Participants were contacted by email and received a link to the survey. The link provided them with information about the study, how their responses would be used, and by starting the survey they gave their consent. The researchers then selected scenarios to be filmed based on survey outcomes and practical constraints, in line with suggestion 3. The first author wrote scripts for the scenarios, aiming at recordings with a duration of 30–70 s, and guided by discussions from police officers, police teachers, and actors. As recommended in suggestion 4, two expert police teachers who also worked actively as police officers read the scenario scripts and were present during recording to ensure realistic recordings. Once the video scenarios were recorded and edited, occlusion points were chosen based on identifying time points when various decisions could be made in line with previous research.

#### 3.1.4 Results

##### 3.1.4.1 Scenario ratings

A full overview of rating data for the 10 scenarios is given in Supplementary material S3. One scenario (S2) was discarded due to a realism rating that was less than 5. An additional scenario (S10) was discarded due to a lack of substantive comments for skill differentiation (i.e., the comments did not provide actionable feedback) and a smaller number of expert ratings (i.e., only 7 out of 9) because there was insufficient reliable feedback to refine and develop the scenario. We then selected the six scenarios for filming based on the overall selection criteria, with greater weight given to scenarios rated higher for their potential to differentiate between skill levels (i.e., skill differentiation). This selection also incorporated the expert police teachers’ feedback as recommended in suggestion 3. A brief description of the selected scenarios is given in Table 1.

**Table 1:** Brief descriptions of the scenarios selected from Part 2 (Production) with labels and sources.

Label	Description	Source
S8: He’s Got a Gun^a^	Domestic disturbance call to apartment with armed, distressed subject along with two others in the apartment culminating in a shooting.	Adapted from Wigsten (2020)
S8: Suicidal Enraged Woman_a_	Suicidal subject verbally confronting the police leading to knife attack on other party and police.	Developed by the first author based on prior research (Suss and Ward, 2012, 2018; Ward et al., 2011)
S5: Man Shouting and Pacing	Police called to the scene with an erratic man pacing and screaming in a parking lot escalating to a baseball bat attack.	Adapted from Suss (2013)
S1: Relationship Abuse	A domestic disturbance in an apartment escalating to a knife attack on partner.	Developed by the first author based on prior research (Suss and Ward, 2012, 2018; Ward et al., 2011)
S7: Car Theft in Progress	Police encounter a suspect with a knife in a parking lot culminating in an unarmed attack on police.	Adapted from Andersen and Gustafsberg (2016)
S6: Domestic Dispute Outside	Police called to a scene of a domestic dispute where a person with a broken bottle approaches and eventually attacks the police officers.	Adapted from Brown and Daus (2015)

a Scenarios not retained in the final measure after testing. Remaining scenarios were included in the final measure. See Figure 2.

##### 3.1.4.2 Scenario production/temporal occlusion

A pictorial overview of the filmed scenarios and occlusion points is given in Figure 2. Each video was temporally occluded (i.e., edited so that the screen turned black) at either two or three time points. The occlusion points were chosen based on when the participant was presented with a decision point (i.e., meaningful stages in the scenario where participants evaluate how they would handle and interpret the situation) in the unfolding event in line with previous research (Mangels et al., 2020; Ta-Johnson et al., 2025). After each occlusion point, participants were asked to generate response options, evaluate the likelihood that each would be used, identify the most important cues, and describe their current understanding of the situation to gain insight into the developing decision-making process in the unfolding scenarios. After the final occlusion point, in addition to the judgments described above, participants were asked to predict what would happen in the next few seconds. Responses were recorded via participants typing them into open-ended text boxes in the measure. The scoring, interpretation, and analysis of these responses were beyond the scope for the current study and are therefore not reported.

**Figure 2:**
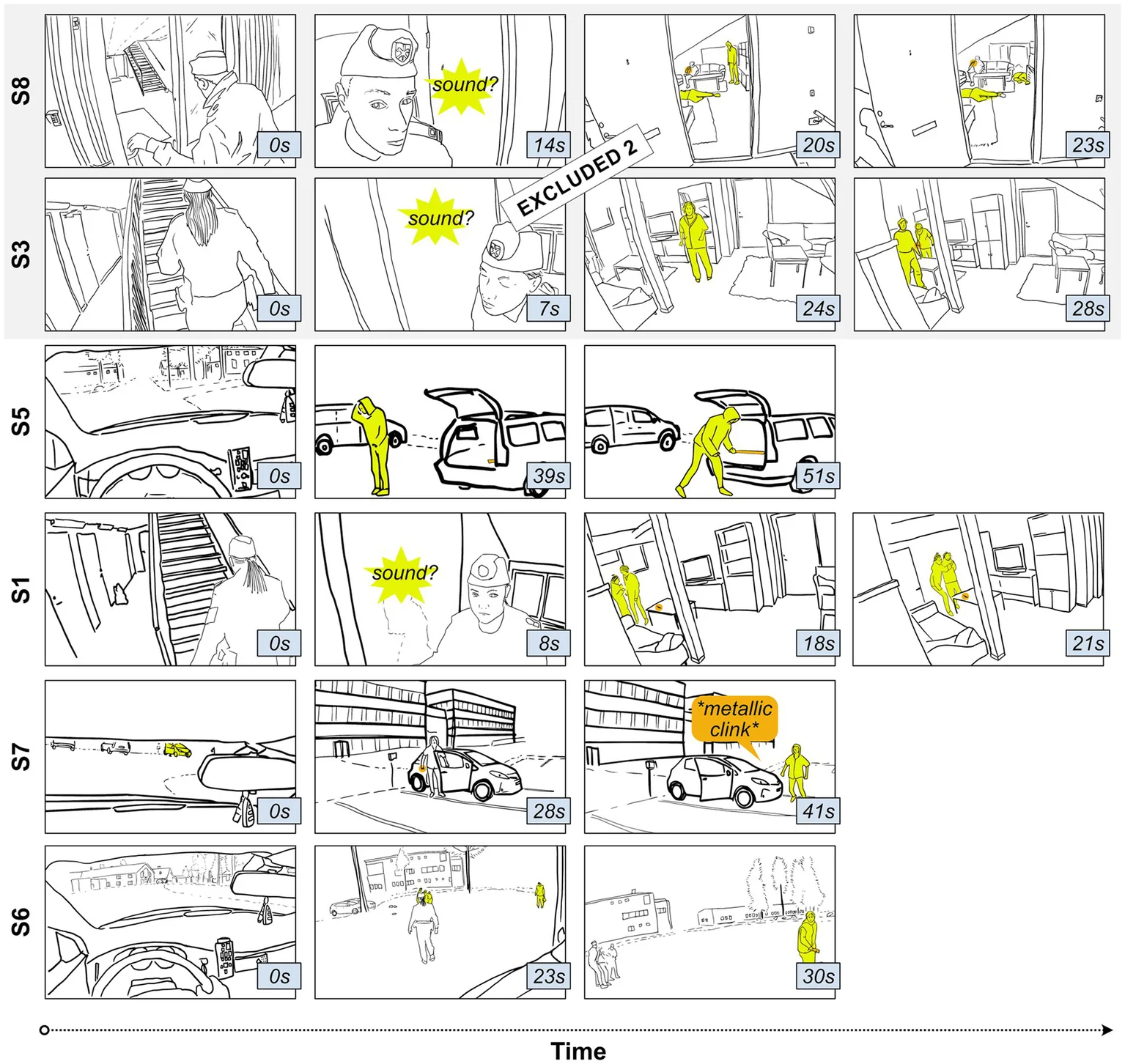
Illustration of scenarios and their corresponding occlusion points from Production (Part 2). For each scenario, the first image displays the first frame of the video. Subsequent images represent the final frame before each occlusion point (i.e., before the screen turns black). The times at the bottom right corner of each image represent the occlusion point times (i.e., in seconds) from the onset of the video scenarios. The final occlusion points in each scenario occurred within 1–3 s before the culminating event. Yellow highlights indicate critical cues. Orange highlights indicate weapon-related cues. The greyed-out scenarios at the top of the bottom panel labeled “Excluded 2” indicate the scenarios that were excluded after pilot testing in Testing (Part 3).

All the videos culminated in a violent attack by the suspect (e.g., punching, swinging a baseball bat). The final occlusion point in each scenario was specifically chosen based on a time point referred to as a critical turning point that was identified (e.g., when a suspect picks up a baseball bat that was lying nearby) that occurred within 1–3 s before the culminating event (i.e., an attack) in line with previous research (Suss and Ward, 2012, 2018; Ward et al., 2011). The final occlusion point was chosen in this manner so that just enough information was provided for the participants to be able to predict the culminating event.

## 4 Part 3: Testing

### 4.1 Method

#### 4.1.1 Participants

Police students in their final year of education (*n* = 8) and Police teachers (*n* = 2) were recruited from Umeå University to pilot test the video scenarios and the procedure as recommended in suggestion 6. The police students and police teachers were recruited by one of the authors of the current study that works as a teacher at Umeå police education.

Participants were recruited through general announcements and invitations. Police students were informed about the study via a course platform, which is routinely used to share research participation opportunities, and no individual students were directly approached by teaching staff. Police teachers were invited via a general email sent to all police teachers.

Participation was completely voluntary. Police students indicated their interest by signing up via an external scheduling tool and police teachers participated after responding to the email invitation. No academic or professional consequences were associated with participation or non-participation. Police students who participated were offered refreshments as a small token of appreciation.

#### 4.1.2 Materials

##### 4.1.2.1 Assessment of recorded scenario

We used PsychoPy (Peirce et al., 2019) and the online platform Pavlovia.org to present the six video scenarios from Part 2 to participants. Following the scenarios, participants were given a short survey that asked them to rate three Likert scale questions from 1 to 7, where 1 was “strongly disagree” and 7 was “strongly agree” along with an open-ended question asking for feedback that could improve the measure. The Likert scale questions were (1) “I experienced the study as strenuous,” (2) “I was able to stay focused throughout the entire investigation,” (3) “I felt stressed during the video scenarios in the study.” The open-ended question asked the participants to give feedback on what could be improved (e.g., if anything was unclear, if it did not work as expected, or in some other way could be improved). This open-ended question was used to evaluate clarity, realism, usability, as well as general aspects of the pilot. Participants were further asked to provide judgments about various aspects of the scenarios for exploratory purposes and were not systematically analyzed in the current study. The exploratory purposes included assessing whether the scenarios allowed varied responses, predictions, and situational buildup (i.e., whether participants had sufficient time and information to perceive relevant cues and understand the situation). Further procedure instructions, question wording, and steps can be found in Supplementary material S4.

#### 4.1.3 Procedure

The students completed the experiment in a computer lab, and the teachers could access the test online by clicking on a link sent to their email. In early pilot testing, participants completed a fixed set of scenarios. In subsequent pilot testing, participants completed three randomly selected scenarios from the full set. Randomization was implemented within the PsychoPy measure using custom code. The final design of the measure involved presenting two randomly selected scenarios, with the option to complete a third randomly selected scenario. The total duration of the procedure was approximately 60 min due to multiple occlusion points per scenario and the requirement for written responses and ratings at each occlusion point.

#### 4.1.4 Results

##### 4.1.4.1 Scenario assessments

**Figure 3:**
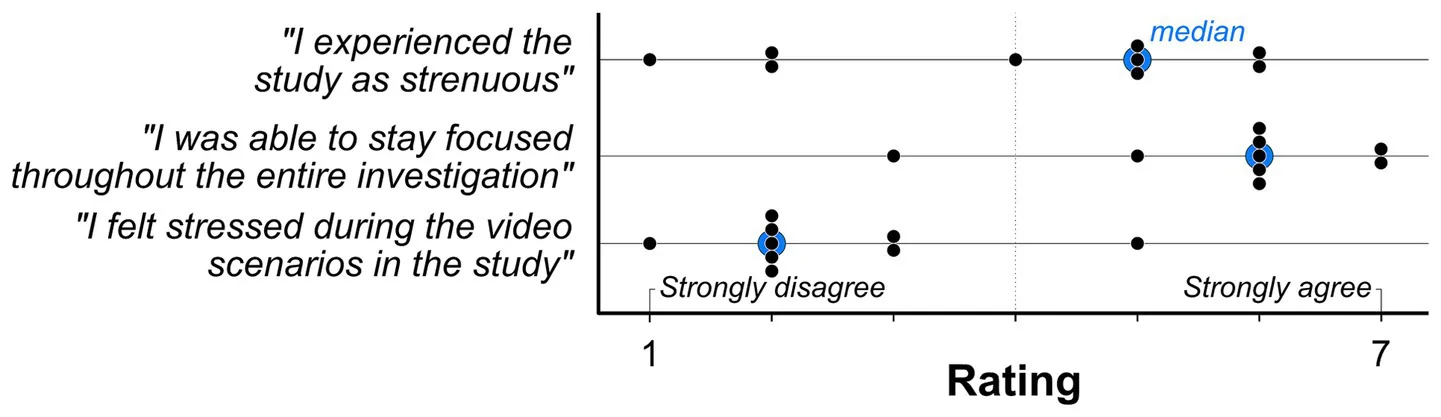
Pilot ratings for the survey items. Each point represents an individual participant’s rating, and the medians are indicated by blue circles. One participant did not complete the survey (*n* = 9).

Participants experienced the measure as moderately strenuous (median = 5, interquartile range = 2–5.5; see Figure 3). Participants reported a relatively high level of focus throughout the measure (median = 6, interquartile range = 5.5–6.5). Reported stress levels during the videos in the measure were low (median = 2, interquartile range = 2–3; see Figure 3). The number of responses per item was *n* = 9.

Across pilot testing, the total number of participants who evaluated each scenario was as follows: S8 (*n* = 6), S3 (*n* = 5), S5 (*n* = 7), S1 (*n* = 6), S7 (*n* = 5), S6 (*n* = 4).

##### 4.1.4.2 Discarded scenarios

Based on the pilot participant responses, two scenarios (S8, S3) from the six produced in Part 2 were excluded from the final measure due to insufficient variation in responses and predictions, as well as limited situational buildup (i.e., insufficient time and information for participants to perceive relevant cues and understand the situation), resulting in a total of four video scenarios (see Figure 2).

## 5 Discussion

In this paper, our aims were to (1) present suggested principles for, and (2) exemplify a three-step method based on temporal occlusion to construct and validate real-world decision-making scenarios. This three-step process offers researchers a structured approach to developing temporally occluded materials and testing them across different domains. The temporal occlusion paradigm has previously shown success in assessing anticipation (Song et al., 2025; Suss and Ward, 2018). This three-step method extracts knowledge from experts to gain a better understanding of the decision-making process that takes place in tasks with varying levels of complexity. It may provide a basis for exploring how expert knowledge could be operationalized into training and policy development and offers guidance on how to (1) systematically select and (2) develop various forms of media that are the most relevant and adapted to the context. This is essential when media such as real videos or professionally produced training films are not available. This process also lays out guidelines on (3) how to pilot test the materials and measure to ensure feasibility, clarity, and a reasonable time duration.

Regarding the extraction step, meeting with domain experts early in the process helps ensure that the research questions and outcome variables that are developed are of interest to both domain experts and researchers. It also assists in determining the most appropriate choice of media and the type of scenarios that are most relevant to the domain of interest. Not consulting with domain experts increases the risk of investing time and effort into materials and methodology that are not relevant or informative to the domain that is being researched.

In the production step, collaboration with domain experts assists in determining the most relevant scenarios that can be used to study decision-making in dynamic, ill-defined, and time-pressured situations. Experts play an integral role in this process by providing feedback on how to improve scenarios and adapt them to the culture being studied. This ensures that the selected scenarios reflect realistic decision-making situations that are encountered in the field. During production of the media (e.g., recording videos, developing virtual reality, etc.), having experts present helps to ensure that the actor’s behaviors (e.g., body language, facial expressions, movements, etc.) and other aspects of the environment (e.g., distance between police and suspect) are as realistic as possible. Experts in other domains will likely choose to focus on different relevant aspects during media production.

Finally, in the testing step, the pilot participants in the current study indicated that the measure was moderately strenuous and that they were able to maintain a high level of focus, suggesting that the task was feasible and engaging. These preliminary results provided support for the feasibility of the task and the scenarios and pointed to possible refinements, including the exclusion of two scenarios that did not work as intended. The pilot also indicated that the participants did not experience the video scenarios as stressful, which is somewhat expected considering the focus on perceptual-cognitive aspects. This may be beneficial because high stress responses could introduce additional factors that would need to be taken into consideration.

Beyond the present findings, this three-step process may be reproduced and adapted to a range of domains. Applied in this way, the resulting measure can be used to gain a better understanding of how experts anticipate future events, select courses of action and weigh the cues and details they see as important when making judgments and decisions. Prior work using temporal occlusion has documented superior expert anticipation across a range of sports (Song et al., 2025) and more recently in law enforcement (Suss and Ward, 2012, 2018), and has linked a more accurate reading of a situation to a higher likelihood of selecting a better course of action (Suss and Ward, 2012, 2018). Temporal occlusion has also been used as a training tool to improve anticipation (Müller et al., 2024; Song et al., 2025), pointing to further applied potential. Our emphasis here, however, is on using the method to understand how skilled practitioners arrive at their decisions. For instance, researchers can investigate whether individuals who apply more appropriate situational strategies within a given scenario achieve better outcomes in terms of anticipation and course of action selection. Future research could investigate similar instruments in other domains.

More recently, the temporal occlusion method has been used with law enforcement focusing on the decision-making differences between experts and novices as well as possible mediators that affect this relationship (Ta-Johnson et al., 2025). The three-step process described here can be adapted for similar studies across domains, and its standardization may facilitate research into how situational and individual psychological factors affect decision-making in dynamic, ill-defined, and time-pressured situations.

## 6 Limitations

There were limitations in this study. The number of experts involved was limited at various steps of this process. However, a small number of experts used for feedback or operationalizing outcome measures is not uncommon in decision-making research using scenarios (Klein and Borders, 2016; Suss and Ward, 2018). The pilot test was likewise based on a small sample and was intended to evaluate feasibility and refine the materials rather than to provide generalizable estimates, and its results should be interpreted with that purpose in mind.

The final edited videos were not formally reevaluated in a systematic way by experts. Although the expert police teachers were involved in the filming process and assisted in making small adjustments to increase realism and how representative the videos were of real patrol work during filming, a formal reevaluation would have strengthened this procedure. There was also no formal review or validation of the selected occlusion points by independent experts after editing. However, one of the scenarios and its occlusion points were presented to a group affiliated with police education, including researchers, police officers, police teachers, and instructors, as part of a presentation, which provided informal feedback.

The described instrument was not empirically validated. Validating whether it performs as intended (e.g., differentiating between skill levels, capturing how experts make decisions in specific scenarios, or to inform decision-making training) falls outside the scope of this study, which concerns the development-and-testing procedure itself. Relatedly, this process was illustrated in one domain, and the transferability of this three-step process has been argued conceptually and not empirically tested. In future studies, this three-step process should be applied to other domains.

Finally, all the scenarios selected for production and included in the final video materials culminated in a violent escalation. Although these scenarios used reflect important, high-risk, dynamic situations in police work, the exclusive focus on escalation may limit the range of situations represented. Future studies could also incorporate de-escalation scenarios to extend the scope of the materials.

## 7 Conclusion

This paper introduces a structured three-step method for developing and testing temporally occluded scenarios. The police example was used and integrated into the process to illustrate how the extraction, production, and testing steps can be implemented. Initial foundational decisions are often not transparent in temporal occlusion research, yet they highly influence subsequent steps and outcome measures. This paper clarifies important aspects that need to be considered such as bringing in domain experts early in the process and using their feedback during development and refinement. This three-step process may be relevant to other domains in which decision-making occurs in dynamic, ill-defined, time-pressured situations, such as paramedics, firefighters, and medical professionals, as well as individuals in leadership roles. This process may also be adapted to different cultural contexts using different media formats. Temporal occlusion has demonstrated that it’s a powerful method that can elicit expert perceptual-cognitive skills, but the process for developing and adapting it to different domains has not been transparent or clear. Our aspiration is that this three-step process lays a foundation for the standardization of temporally occluded scenario development, enabling broader use in research and training contexts.

## Data Availability

The materials supporting the conclusions of this study are provided in the Supplementary material, including detailed scenario descriptions, scripts, occlusion timings, and procedural steps. The original video recordings are not publicly available due to ethical and privacy considerations, as participants consented to their use for research and educational purposes but not for public dissemination. Further information may be made available by the corresponding author upon reasonable request, subject to ethical approval.
