# Supplementary material for: Developing and preliminary testing of temporal occlusion decision scenarios in dynamic contexts: a three-step method
Source: Front Psychol. 2026 Aug 11;17:1893159. doi: 10.3389/fpsyg.2026.1893159 (PMC13503250; doi:10.3389/fpsyg.2026.1893159)
Supplement: Supplementary file 1 [file Data_sheet_1.pdf]

Supplementary Material

Supplementary Material S1

S1.1 Scenario Descriptions Translated into English (n = 10)

- **S1: Relationship Abuse** (Developed by the first author based on prior research (Suss & Ward, 2012, 2018; Ward et al., 2011) and discussions with police teachers) – Police are called to an apartment where a man and woman are reported to be screaming. Once the police enter the apartment, they see a man yelling at a woman and threatening to kill her. Once the man yelling notices the police, he lightly shoves the woman to the side and lunges towards a knife that is lying on the coffee table in the living room. The man picks it up and holds it toward the woman for a short time and eventually decides to stab the woman.
- **S2: Location Search** (Adapted from Ward et al. (2011)) – Police are called to an apartment where a woman has called the police. The woman who called the police has locked herself in the bathroom and feels threatened by a man. There was another woman present in the apartment as well, but the woman is not sure if the other woman and man are still in the apartment. She says that the man has threatened her with an axe. When the police approach the apartment, they see that the door is slightly ajar. They stop and listen but do not hear anything and decide to enter the apartment. When police enter the apartment to have a look and conduct a location search, the suspect hears that the police enter and looks out from a room he is in. He becomes very upset and starts screaming as he goes back into the room. The police hear a woman's voice from the bathroom pleading for help and saying that the man threatened to kill her. The officers begin moving defensively towards the room the man is in. Once there, they see a table with an axe on it and the suspect is standing in the corner of the room with a gun pointed towards a woman's head. After a few seconds, the suspect gives up, drops the weapon, and releases the hostage. Immediately after the hostage is released, the suspect pulls another firearm from his waistband and fires in the direction of the police.
- **S3: Suicidal Enraged Woman** (Developed by the first author based on prior research (Suss & Ward, 2012, 2018; Ward et al., 2011) and discussions with police teachers) – Police are dispatched to an apartment because someone is worried about the person. Once the police arrive at the apartment, they pause for a moment to hear what is happening. They can hear some screaming from the hallway outside the apartment, but they can't make out what's being said. Once the police go in, they find the woman acting very erratic and arguing with a man. It looks as though the woman has a knife. There is a man lying on the floor bleeding from the stomach. Once the woman notices the police, she begins to become verbally aggressive. She moves slowly towards the police and then turns around and walks away. As the woman walks away further into the living room, the man begins to slowly walk out of the room to let the police handle the situation. Suddenly, the woman pulls out a knife that she has concealed and begins to move towards the man and stabs him in the back and then begins moving towards the police.
- **S4: Disturbance in the Home** (Adapted from Suss et al. (2014)) – The police are dispatched to a home where there is a disturbance. When the police arrive outside the apartment, they hear some arguing but can't make out what's being said. Suddenly, they hear a loud bang followed by the sound of a woman screaming in pain. The police enter the apartment, and

they see a man in the room and a woman sitting on the couch crying in front of the man. The man is yelling at the woman and making gestures with his hands. When the woman notices the police, she gets up and says that the man hit her and leaves the room. Then the man turns towards the police, takes a step towards them and starts talking to the officer while gesturing with his hands. The man begins moving slowly toward the police but then turns around and walks in another direction. The man then takes a step toward the police telling them that they shouldn't be there while gesturing with his hands. The man takes another step toward the police again with his right fist clenched, pulls his fist back to shoulder height and punches the police officer.

- **S5: Man Shouting and Pacing** (Adapted from Suss (2013)) – Police are called to a parking lot where a man was reported to be acting irrationally. Once police arrive, they see a man near a car in the parking lot. The car's front doors and the hatchback are open. The man is pacing back and forth talking loudly to himself and looks upset. One of the police officers goes to tell some spectators that are nearby to leave the scene and asks the other police officer to take care of the man. When the man sees the police officer approaching, he becomes verbally aggressive. As the police officer comes closer to the car, he notices that a baseball bat is in the hatchback of the car. The man suddenly takes a step towards the car and leans into the hatchback and pulls out a baseball bat. Then he starts moving towards the participant while raising the baseball bat over his shoulder as if preparing to swing at the police officer. Then the man attacks the officer with the baseball bat.
- **S6: Domestic Dispute Outside** (Adapted from Brown and Daus (2015)) – Police are dispatched to a field near the house of the person who called in the disturbance. Once the police arrive on the scene, there are two police in the process of trying to get a man under control that is verbally and physically aggressive but appears unarmed. There is also a woman seen standing in the area. The police that just arrived start moving towards the two police to help them when all of a sudden, the woman becomes angry and begins to move towards the police. She pulls a broken bottle out from a bag she has and begins brandishing it towards the police and ignores commands. She moves quickly towards the police and after a short time attacks the police with the broken bottle.
- **S7: Car Theft in Progress** (Adapted from Andersen and Gustafsberg (2016)) – Police are called to a parking lot where a man was reported to be searching through cars. When police arrive at the scene, they see a man that is leaning into a car. Suddenly, the man gets out of the car and sees the police. He stands up by the car door with a knife in his hand. Shortly after the man begins to quickly move to the other side of the car. On the other side of the car, he drops the knife and then proceeds to quickly walk towards the police with clenched fists. As he's moving towards the police, he pulls his hand back and punches the officer.
- **S8: He's Got a Gun** (Adapted from Wigsten (2020)) – Police are called to a residence where there were reports of yelling and loud banging. Once the police arrive outside the door to the apartment, they hear a loud sound coming from the apartment. The police open the door to the apartment and enter. The loud sounds are coming from the room to the right. The police approach the room to investigate what is happening. The police look into the room and see a man sitting in a chair in the corner of the room pointing a gun at his head. The man is not communicative and is staring at the floor. The police also see a non-responsive man on the ground that seems to have been shot. There is also another person in the room that is pacing back and forth. The pacing man is not communicating and seems to be in shock. The suspects don't react to anything the police say. After a short time, the man with the gun in the chair starts to slowly move the gun towards the shocked man pacing. After a short time, he shoots

the shocked man and starts to point the gun towards his head again. After a few seconds, the man shoots himself in the head and falls to the ground.

- **S9: A Fight Breaks Out After a Soccer Match** (Developed by the first author based on prior research (Suss & Ward, 2012, 2018; Ward et al., 2011) and discussions with police teachers) – Police are dispatched to a field not far from a soccer stadium after a match where two men are arguing loudly and appear to be intoxicated. When the police arrive on the scene, they see two men arguing and begin to physically fight. The officers approach the two men, but the men ignore what the police say and keep fighting. After a few seconds, one of the men turns around and starts telling the police to leave. Then the man bends down and picks up a rock from the ground and throws it at one of the police officers. The rock hits the officer in the chest. Then the suspect picks up another rock and pulls his arm back to shoulder height and throws the rock at the officer.
- **S10: Fight Outside a House** (Developed by the first author based on prior research (Suss & Ward, 2012, 2018; Ward et al., 2011) and discussions with police teachers) – Police are called to a disturbance in a home. The caller reported that her husband is standing outside their home and banging on the house's window. The man is upset and angry. When the police arrive, they see the man in the middle of the street that matches the description of the person. The suspect is walking down the street toward the police officers. The police officers slowly approach the man and notice that he seems intoxicated. When the officers are closer to the man, they notice that he has an axe in his hand. The man ignores all commands from the police and continues to slowly walk forward. After a few seconds, the man makes eye contact with the police and slowly lowers the axe to the ground. As the police move towards the man to arrest him, the man suddenly pulls a knife from his pocket and lunges at the police officer with it and stabs the officer.

S1.2 Survey Rating Items

We created a survey in the Swedish language in Microsoft Forms with ten scenario descriptions that culminated in violent escalations accompanied by the following questions translated into English (open-ended questions were displayed under the Likert scale questions):

- Likert scale question evaluating how realistic the scenario was: "How realistic do you think the scenario is? Indicate on a scale from 1 – 10, where 1 is poor and 10 is excellent."
    - Open-ended question: "Is there anything you think could be changed to make the scenario more realistic?"
- Likert scale question evaluating police decision-making skill level discrimination: "To what extent do you feel that the scenario provides opportunities to distinguish skill levels (i.e. to what extent does the set-up of the scenario help to enable different decisions to be made that will differentiate between officers with high skill/experience and those with less skill/experience? Indicate on a scale from 1 – 10, where 1 is poor and 10 is excellent."
    - Open-ended question: "Is there anything you think could be changed in the scenario to increase the likelihood that it could help differentiate between officers with different skill/experience?"

S1.3 Complete Analysis decision rules

The following section describes the operational decision rules used during the scenario evaluation analysis more specifically.

In the current study the analysis was conducted in two phases, where the first phase evaluated if the scenarios mean realism ratings were over a threshold for moderately realistic (e.g., mean ≥ 5) and those that failed to meet this threshold did not advance to the next phase. This strategy was chosen because the aim of this survey was to develop realistic scenarios that were able to differentiate between skill levels, so skill differentiation was weighed more heavily because the measure will be used to distinguish decision-making skill levels in future studies. In addition, for each scenario that was deemed to be moderately realistic and faithful to situations encountered in the field, the comments for realism improvements may improve this aspect and were unlikely to lower these ratings.

The second phase of the analysis evaluated the remaining scenario description ratings on skill differentiation where ratings were ranked based on highest to lowest means ratings. In addition to the rankings in phase 2, other aspects were taken into consideration such as the range of variation between the scenarios (e.g., suspect gender, age and size, mix of parked vehicles), systematic differences between the scenarios (indoor vs. outdoor context, presence vs. absence of bystanders, presence vs. absence of potential victim with suspect), the participants understanding of what was trying to be achieved, and previous research. Some of the comments indicated that some of the participants at times didn't understand the aim of the evaluation (e.g., rated the skill differentiation aspect based on what the officers could do after the outcome, reasoning based on basic police education, or that the scenario was too difficult). These ratings were excluded when the data was cleaned. In addition, we prioritized scenarios that had at least 2 substantive comments for skill differentiation when selecting scenario descriptions for recording. The comments for improvement provided in the open-ended questions that were relevant and feasible were incorporated into the scenarios as much as possible.

Supplementary Material S2

S2.1 Script development

The scripts were written by the first author. The structure and realism were guided by police officers and police teachers and informed by reviewing scenario scripts used in Swedish police education for scenario training with actors, consulting publicly available script examples, and obtaining informal feedback from a local actor with some acting experience. The movie scripts specified each role's actions, verbal communication, body language, emotional expression, as well as relevant environmental details. Scripts were refined using participant feedback from the survey comments. Six paid actors were hired and six police students participated voluntarily. Two expert police teachers who worked as police, with scenario training experience, reviewed the scripts and provided on-site guidance to support realism/ecological validity.

S2.2 Script Examples Translated into English

The following script examples are the scripts that were distributed to actors, police students, and police teachers before filming. The scripts reflect the structure, critical cues, and actions. During filming, small adjustments (e.g., distance, timing, intended behaviors or actions) were made that were

guided by the two experienced police teachers that were on-site. These adjustments were made to improve realism and support high external validity, not to alter the logic of the scenarios.

S1: Relationship Abuse

Conditions

Two police officers are on Patrol (Police Officer 1 and Police Officer 2); Police Officer 1 is the lead officer (the one wearing the body camera), and Police Officer 2 is their partner. The officers receive the call over the radio.

Radio call/ Context

The Police are called to a reported disturbance in a residence. A neighbor reported they heard thuds, screams, and breaking glass coming from the neighboring apartment. The name on the door is Larsson and it's on the first floor. According to the caller, the front door is ajar. There is no prior history at this address.

Film Script:

What is needed:

- 2 actors: A man and a woman
- 2 police students
- 1 or 2 police instructors to ensure the scene is filmed with high quality
- Knife

Instructions to Actor 1 (the man)

You are very angry at your partner, the woman. You are yelling at her and are close to losing control. When you notice that Police Officer 1 is looking at you, you become frightened and immediately angry, and you move very quickly toward a knife lying on the table about a meter away from you. Once you have picked up the knife, you grab the woman, stand behind her (with two-thirds of your body visible), and press the knife against her side. As you stand behind the woman, you look angry and desperate. After 3–5 seconds, you stab the woman and she falls to the ground.

Instructions for Actor 2 (the woman)

You are arguing with your partner, the man. You try to de-escalate the argument with the man. You speak submissively while he yells. When you see the police, you are surprised but relieved that they are there. But before you can do anything, the man grabs you and has picked up the knife, which he presses against your side. You are very scared and shocked. The man eventually stabs you in the side, and you fall to the ground.

Enter the apartment building

Police officer 1 and Police Officer 2 open the front door to the apartment building and enter the building through the door.

Inside the apartment building

The police officers walk up the stairs to the first floor. When they reach the first floor, they begin to hear screams clearly coming from one of the apartments. As the officers approach the door to the apartment where the screams are coming from, they clearly hear someone arguing a little further inside the apartment. The police officers notice that the door is slightly ajar and stand there for a moment to hear what is going on.

Woman

Please, I’m not lying. Calm down

Man

You should be damn grateful that I put up with you. Do you get that?

Woman

In a somewhat submissive tone: We can talk about it. It’s okay

Man

Shut up! Say that again and I’ll kill you!

Police Officer 1 decides to open the apartment door and sees the entryway in the apartment and notices that the arguing is coming from the kitchen, which is located about 4 meters to the left from the entryway to the apartment. Police Officer 1 cannot yet see the individuals.

Police Officer 1 decides to enter the apartment and Officer 2 follows. Police Officer 1 walks toward the kitchen, and as soon as the arguing couple comes into view, the man grabs a knife lying on the table about a meter away. The man suddenly grabs the woman standing next to him, positions himself behind her, and holds the knife against her side. Two-thirds of the man’s body is exposed as he stands behind the woman.

After about 3-5 seconds, the man stabs the woman in the side, and she falls to the ground.

S5: Man Shouting and Pacing

Conditions

Two police officers are on Patrol (Police Officer 1 and Police Officer 2); Police Officer 1 is the lead officer (the one wearing the body camera), and Police Officer 2 is their partner. The officers receive the call over the radio.

Radio call/ Context

The police receive a call reporting that a man is acting strangely in the parking lot outside of the caller’s home.

Film Script

What is needed:

- 3 actors: A man; two others to play bystanders near the scene

- 2 police students
- 1 or 2 police instructors to ensure the scene is filmed with high quality
- Private car
- Baseball bat
- Police car

Instructions for Police Officer 1

You park the police car about 10 meters from the person and get out of the police car. You move slowly toward the person together with your partner, Police Officer 2. The two of you notice that there are a few people nearby whom you want to remove from the area. Your partner, Police Officer 2, decides to tell the people to leave the area and asks you to handle the man. As you approach the man, he becomes verbally aggressive. When you are about 4–6 meters from the man, he reaches into the car's hatchback and grabs a baseball bat. You say or do nothing but simply stand and watch as the scenario unfolds. The man begins moving toward you with the baseball bat, prepares to swing it, and then swings the baseball bat at you.

Instructions to Police Officer 2

You step out of the police car after it has been parked. As the two of you walk toward the man (about 9 meters from him), you say that you will tell the people to leave the area and ask Police Officer 1 if he can handle the man. You walk to the people to tell them to leave the area while Police Officer 1 approaches the man.

Instructions for Actor

You will look paranoid and as if you are struggling with something mentally. You will argue with yourself under your breath while making facial expressions and hand gestures as if you were arguing with another person. When you see Police Officer 1, you become verbally aggressive and start shouting. When Police Officer 1 is about 4–6 meters away, you grab a baseball bat that is in the hatchback of the car and start taking steps toward Police Officer 1 while raising the baseball bat above your shoulder as if you're preparing to swing at Police Officer 1. Then you swing the baseball bat at Police Officer 1.

In the parking lot

Police Officer 1 and Police Officer 2 locate the man next to the car in the parking lot. The police officers park their car about 10 meters from the car and get out. The car's front doors and hatchback are open. The man appears to be arguing with himself and looks agitated. The man is standing about half a meter from his car. Police Officer 2 moves the other bystanders away from the area (i.e., the bystanders by the car) and asks Police Officer 1 to handle the man. When the man sees Police Officer 1, he begins to become verbally aggressive.

Man

Don't mess with me. I don't trust you. Stay away from me.

As Police Officer 1 begins to approach the man, they notice a baseball bat in the car's hatchback. The man then quickly takes a step toward his car and grabs the baseball bat from the car. Police Officer 1 is approximately 4–6 meters from the car. The man then begins moving toward Police Officer 1

while raising the baseball bat above his shoulder as if preparing to swing at Police Officer 1. The man then swings the baseball bat at Police Officer 1.

S6: Domestic Dispute Outside

Conditions

Two police officers are on Patrol (Police Officer 1 and Police Officer 2); Police Officer 1 is the lead officer (the one wearing the body camera), and Police Officer 2 is their partner. The officers receive the call over the radio.

Radio Call/Context

The police received a call from an anonymous caller reporting that a couple is arguing outdoors. The police are dispatched to the argument outdoors near the caller’s street (at Hallongränd 6).

Film Script

What is needed:

- 2 actors: A man and a woman
- 4 police students
- 1 or 2 police instructors to ensure the scene is filmed with high quality
- Broken bottle
- Handbag
- Police car

Instructions for actor 1 (the man)

You become verbally aggressive and angry because the police are there. You argue with the police when Police Officer 1 and Police Officer 2 arrive at the scene.

Instructions to actor 2 (the woman)

Before Police Officer 1 and Police Officer 2 arrive, you were arguing with your boyfriend, Actor 1, when a police patrol arrived and broke up the argument. Actor 1 became verbally aggressive and angry at the police when they arrived, so they were busy dealing with him. You stand a short distance away while the first officers on the scene and actor 1 are arguing.

When Police Officer 1 and Police Officer 2 arrive at the scene, you are still standing a short distance away while the police patrol and Actor 1 are arguing. You are upset but appear calm. Suddenly, you become furious. You start moving towards Police Officer 1, and when Police Officer 1 sees you, you take the broken bottle out of your handbag, drop the handbag, and begin waving the broken bottle at Police Officer 1 when you are 3-4 meters away from Police Officer 1. After a few steps, you are close to Police Officer 1 and strike Police Officer 1 with the bottle.

Outdoors in the field next to a street

When the police officers arrive at the scene, there are already two officers there confronting the man, who is verbally aggressive but appears to be unarmed. The woman stands a short distance from the police patrol arguing with the man and watches. Police Officer 1 and Police Officer 2 rush toward the two officers to assist them, but suddenly the woman charges furiously toward Police Officer 1. Out of nowhere, she pulls a broken bottle from her handbag and begins swinging it. Then the woman drops her handbag and finds herself about three to four meters from Police Officer 1. After a few seconds, she strikes Police Officer 1 with the broken bottle.

S7: Car Theft in Progress

Conditions

Two police officers are on Patrol (Police Officer 1 and Police Officer 2); Police Officer 1 is the lead officer (the one wearing the body camera), and Police Officer 2 is their partner. The officers receive the call over the radio.

Radio Call/Context

The police are called to an area where a man is searching through cars in a parking lot.

Film Script

What is needed:

- 1 actor: A man
- 2 police students
- 1 or 2 police instructors to ensure the scene is filmed with high quality
- Private car
- Knife
- Police car

Instructions for Actor 1

You will be armed with a knife. You are a drug addict that will look somewhat desperate as you search for money to get your next fix. You will be mentally ill and perhaps even in a state of psychosis. You will lean into the car with one knee on the passenger seat to search through it. You will look surprised when you see the police officer. Once out of the car, you will look confused and scared while holding the knife in your right hand. Suddenly, you become angry and start running around the back of the car to the other side of the car. You go to the other side of the car, drop the knife, and start running around the front of the car. You jog toward Police Officer 1, and with clenched fists, you ignore everything Police Officer 1 says and then pull your hand back to punch Police Officer 1. Then you punch Police Officer 1.

In the parking lot

Police officer 1 and Police Officer 2 arrive at the scene where the man is searching through a car. The man is searching through the car on the passenger side of the car when the police see him. A backpack lays on the ground behind the car. Police Officer 1 is about 6 meters from the car and

begins to approach the passenger side of the car the man is searching through. Suddenly, the man looks up and sees Police Officer 1 when Police Officer 1 is 5-6 meters away. The man steps out of the car, stands up with a knife in his hand, and quickly runs around the back of the car to the other side of the car. On the other side of the car, the man drops the knife and then continues running around the front of the car to the passenger side again toward Police Officer 1 with clenched fists, ignoring everything Police Officer 1 says, and then pulls his hand back to punch and punches Police Officer 1.

S2.3 Filming procedures and technical information

The videos were recorded in first-person perspective with a Go Pro camera mounted on one of the police students' heads during each film. The videos were recorded at 1080p at 60 frames per second. Six paid actors were hired to portray suspects, victims, and bystanders, and six police students participated voluntarily to portray the police officers in the video scenarios. Informed consent to be recorded in the video scenarios was obtained from the actors and the police students before filming. The police students were in police uniforms and equipped with imitation weapons that are used in training. The actors and police students that acted in each scenario were varied. Each scenario was filmed three or four times to increase the likelihood of producing quality videos by providing opportunities to clip together different segments of the videos. For the videos that were filmed inside, filming lights were set up to increase the filming quality. Props such as knives, alcohol bottles, weapons, and police cars were used in the videos.

S2.4 Exact Timing of Occlusion Points and Culminating Events for Each Selected Scenario

Supplementary Table S2.1. Timing of occlusion points, culminating event, and time between final occlusion point and culminating event for each scenario.

Scenario	Occlusion Point 1 (s)	Occlusion Point 2 (s)	Occlusion Point 3 (s)	Culminating Event (s)	Time Interval between Final Occlusion Point and Culminating Event (s)
S8: He's Got a Gun*	14	20	23	24	1
S3: Suicidal Enraged Woman*	7	24	28	30	2
S5: Man Shouting and Pacing	39	51	NA	54	3

S1: Relationship Abuse	8	18	21	24	3
S7: Car Theft in Progress	28	41	NA	43	2
S6: Domestic Dispute Outside	23	30	NA	33	3

Note. Times are given in seconds from the onset of each scenario. “Time Interval between Final Occlusion Point and Culminating Event” refers to the time interval (i.e., in seconds) between the final occlusion point and the culminating event in each scenario. *Scenarios not retained in the final measure after testing. Remaining scenarios were included in the final measure.

S2.5 Video Editing

After the video scenarios were recorded, they were edited in Adobe Premiere Pro to alter the color, mask certain parts out to increase realism, and clip the videos at the occlusion points. The measure was made in PsychoPy version 2024.2.3 (Peirce et al., 2019) and ran on Pavlovia.org by clicking on a link.

Supplement Material S3

The following figures show the full and cleaned datasets for Realism and Skill differentiation from Part 2: Production.

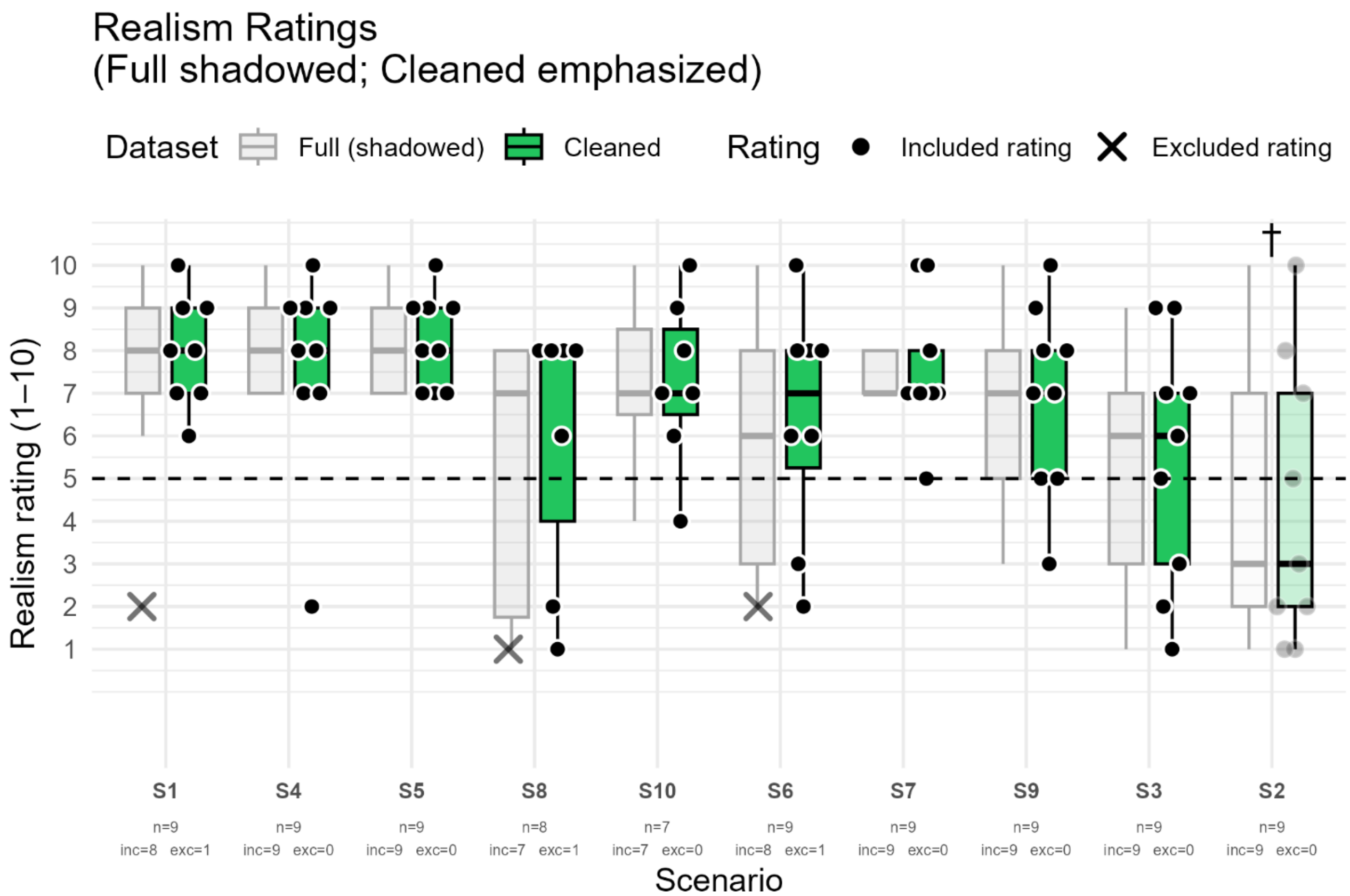

S3.1 Figure S1. Grey (shadowed) and green boxplots show the full and cleaned datasets for realism, respectively. Boxes represent the interquartile range, whiskers extend to the most extreme values within 1.5 x IQR, and the short horizontal bar indicates the median. Black points represent included ratings and "X" represents excluded ratings. Scenarios are ordered by the cleaned median realism rating. The "†" indicates scenarios below threshold. The only scenario that had a mean realism rating below a 5 was S2. Under the scenario numbers, "n" indicates the total number of participants that rated the scenario, "inc" is the number of ratings that were included, and "exc" is the number of ratings that were excluded.

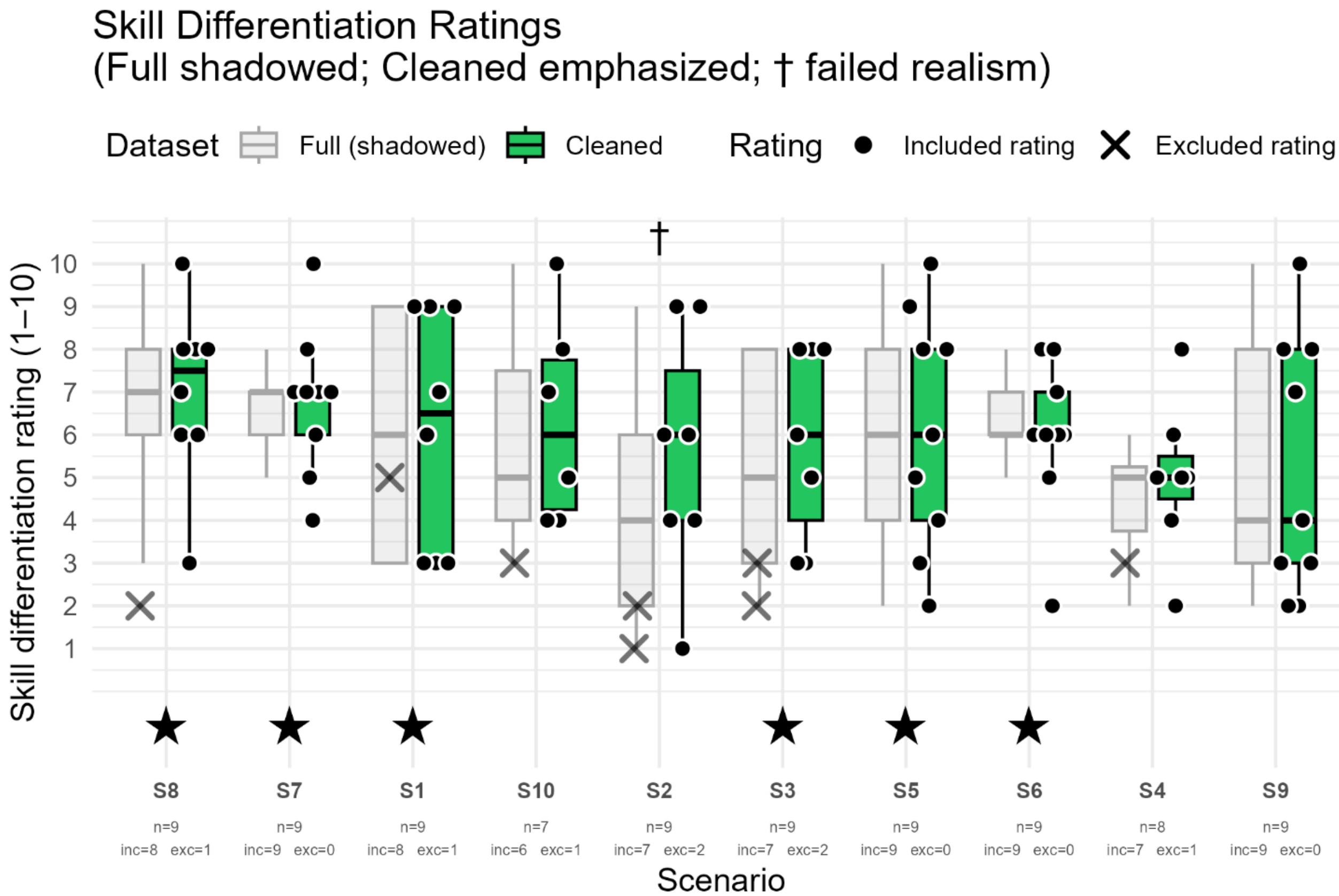

S3.2 Figure S2. Conventions are the same as in Figure S1. Scenarios are ordered by the cleaned median skill differentiation rating; ★ indicates the scenarios selected for recording and † indicates scenarios that failed the realism threshold.

Supplement Material S4

S4.1 Complete instructions

The procedure instructions were originally in Swedish and have been translated into English. The participants were presented with the following instructions. In this study, you will watch video clips of police situations. The videos will pause at predetermined time points. At each pause, you will be asked to assess the situation, suggest different courses of action for how you, as a police officer, would handle the situation, and at times predict what will happen next. You will also be asked to explain your reasoning behind the different courses of action.

You will begin with a practice round to familiarize yourself with the format by watching a video clip and answering questions about it. During the practice round, it may be helpful to type a few simple courses of action to see how the process works, including how you rate the options.

In the study, you will then be shown two video scenarios and have the opportunity to complete a third. Each video will pause at two or three points. When the video pauses, we want you to write down the action options that you considered when the video paused or immediately afterward, without considering the likelihood that you would use them. After you have listed the possible actions, you will estimate how likely you are to perform the decisions.

S4.2 Question wording and step by step procedure

Stage 0: Practice Trial

The measure started with welcoming the participant, explaining how the procedure worked and starting with a practice trial (practice trial used a scenario adapted from Mangels et al. (2020)) to familiarize the participant with the procedure and reduce confusion. The practice trial followed the full sequence of steps for one scenario. The participants began the procedure with a practice video scenario that showed all the steps of the measure, and they could repeat the practice trial as many times as they wanted.

Stage 1: Briefing Phase

Each scenario began with a short text briefing describing the situation to which the officers were dispatched. The briefing contained very brief information about why the officers were called to the situation and what initial information was known about the suspect(s) and/or the victim(s) and the situation. The participants were asked to describe their initial thoughts about the situation based on the briefing by typing them into a text box.

Stage 2: First Occlusion Point Response Generation and Likelihood Ratings

Next, they were shown a segment of one of the selected video scenarios up to the first occlusion point (i.e., when the video was paused and the screen turned black). After the occlusion point, participants answered open-ended questions by typing their answers into text boxes. First, participants listed plausible response options (i.e., courses of action) that they were considering when the video stopped or immediately afterwards based on what they had seen in the video and the initial briefing. They were instructed to write response options, regardless of the likelihood of using them. Participants were provided with six text boxes (open-ended) arranged in a 3 x 2 layout (i.e., two columns x three rows of text boxes) below the question to write their answers. The text boxes had no character limits and filling them in was optional to proceed. The question used for eliciting the response options was adapted from Suss and Ward (2012, 2018) and stated "Based on the information you had when the video was stopped, how could you handle the situation (what could you do) in the next few seconds? You can describe different courses of action (however, you don't need to fill in all the boxes below)."

After the participants wrote their response options, the response options were presented back to the participants in a random order with Likert scales underneath each text box, and they were instructed to rate the likelihood of using each option from 1: "not at all likely" to 10: "very likely" (adapted from Suss and Ward (2012, 2018)).

Stage 3: Rationale, Cues, and Sensemaking Probes

Rationale Prompt. Next, the participants were shown their response options along with their ratings and were asked to give a rationale which explained the reasoning behind their response option ratings (Klein & Borders, 2016). The prompt for the rationale used in the current study was "Please explain your reasoning when you rated the different options."

Cue Identification. After entering their rationale, the participants were asked to describe the three most important cues they heeded in the situation at the occlusion point. The probe used for identifying the cues heeded in the current study was "From the time that the video started until it stopped, which factors/cues did you pay most attention to (something you saw, heard, or otherwise noticed)? List the three factors/cues that you feel were most significant and describe their importance."

Sensemaking/ Situation Assessment. Next, the participants were asked about their sensemaking of the situation at the occlusion point, also referred to as situation assessment. The probe used for understanding the sensemaking of the situation was "Based on the information you have received so far about the situation and the video; how would you describe what is happening right when the video stops?". These open-ended questions for cue identification and sensemaking were created from common probes used in cognitive task analysis (adapted from Crandall et al. (2006); Mangels et al. (2020)).

Stage 4: Subsequent Occlusion Cycles

This sequence of steps described above was repeated for each occlusion point in each of the scenarios evaluated.

Stage 5: Final Occlusion Point Prediction Phase

After the final occlusion point in each scenario, in addition to the other questions above, participants were asked what they thought would happen next in the scenario (Suss & Ward, 2012, 2018). The question used in the current study was "Based on the information you had when the video stopped, what do you think will happen in the next few seconds? You can describe different possibilities (you do not need to fill in all the boxes below)". There were six boxes set up below the question in the same way as the response options (i.e., in a 3 x 2 layout). After the participants entered their predictions about what they believed would happen, their prediction options were presented back to them in random order in the same layout as the response options. The participants were instructed to rate the likelihood of each option occurring next from 1: "not at all likely" to 10: "very likely" (adapted from Suss and Ward (2012, 2018)).

Stage 6: Full Video & Evaluation of Final Occlusion Point Timing

Viewing the Full Scenario. Finally, the participants were told that the scenario they were just shown was stopped before a culminating event (e.g., an attack by the suspect) and that they would soon be shown the full scenario with the final pre-selected occlusion point that was just before a culminating event. Before the full scenario was presented, the participants were asked to evaluate if the final pre-selected occlusion point in the video was placed at a time point where they believed there was enough information for a police officer to be able to predict the culminating event or if they believed that the event could have been predicted at an earlier or later point in the video. Then the participants were presented with the full video with the pre-selected occlusion point (i.e., shown with a 3 second pause) and the culminating event.

Evaluation of Final Occlusion Point Timing. The question used after the full video presentation was "Was the video paused at a time when you think it is possible for a police officer to predict the culminating event?" and they answered with "yes" or "no". If the participant selected "yes", then the scenario evaluation trial was completed. If the participant chose "no", they were

asked if they wanted to change the pause time to an earlier or later time point in the video so that it is possible for a police officer to predict the culminating event. The question used in the current study was "Do you want to change when the video was paused so that it is possible for a police officer to predict the culminating event" and the participants answered with "yes" or "no". If the participant chose "no" the video scenario evaluation trial was completed

Adjusting the Final Occlusion Point. If the participant chose "yes" to changing the final occlusion point then they were brought to a screen where the scenario was displayed in a paused state 10 seconds before the pre-selected occlusion point was first presented (i.e., the pre-selected occlusion point was not shown in this step). In this step, the participant had the ability to choose the time point (i.e., occlusion point) where they believed the video should be paused to fit the criteria. In the measure, the time point saved was the frame number based on the frames per second FPS of the video.

When the participants were presented with the paused video, there were instructions at the bottom of the screen guiding them in how they could start the video, pause the video, rewind, and confirm the pause time they chose. After the participants chose a new pause time, they were presented with the video scenario with their chosen pause time and asked if they were satisfied with their pause time selection. Participants could repeat this process until they were satisfied with the pause time they chose. Once the participant was satisfied with their chosen pause time, the video scenario evaluation trial was completed.

References

Andersen, J. P., & Gustafsberg, H. (2016). A Training Method to Improve Police Use of Force Decision Making: A Randomized Controlled Trial. *Sage Open*, *6*(2), 2158244016638708. https://doi.org/10.1177/2158244016638708

Brown, S. G., & Daus, C. S. (2015). The influence of police officers' decision-making style and anger control on responses to work scenarios. *Journal of Applied Research in Memory and Cognition*, *4*(3), 294-302. https://doi.org/10.1016/j.jarmac.2015.04.001

Crandall, B., Klein, G., & Hoffman, R. R. (2006). *Working minds: A practitioner's guide to cognitive task analysis* [doi:10.7551/mitpress/7304.001.0001]. Boston Review. https://doi.org/10.7551/mitpress/7304.001.0001

Klein, G., & Borders, J. (2016). The ShadowBox Approach to Cognitive Skills Training: An Empirical Evaluation. *Journal of Cognitive Engineering and Decision Making*, *10*(3), 268-280. https://doi.org/10.1177/1555343416636515

Mangels, L., Suss, J., & Lande, B. (2020). Police Expertise and Use of Force: Using a Mixed-Methods Approach to Model Expert and Novice Use-of-Force Decision-Making. *Journal of Police and Criminal Psychology*, *35*(3), 294-303. https://doi.org/10.1007/s11896-020-09364-4

Peirce, J., Gray, J. R., Simpson, S., MacAskill, M., Hochenberger, R., Sogo, H., Kastman, E., & Lindelov, J. K. (2019). PsychoPy2: Experiments in behavior made easy. *Behavior Research Methods*, *51*(1), 195-203. https://doi.org/10.3758/s13428-018-01193-y

Suss, J. (2013). *Using a Prediction and Option Generation Paradigm to Understand Decision Making* [Doctor of Philosophy in Applied Cognitive Science and Human Factors, Michigan Technological University]. Houghton, Michigan. https://digitalcommons.mtu.edu/etds/495

Suss, J., Belling, P., & Ward, P. (2014). Use of Cognitive Task Analysis to Probe Option-Generation in Law Enforcement. *Proceedings of the Human Factors and Ergonomics Society Annual Meeting*, *58*(1), 280-284. https://doi.org/10.1177/1541931214581058

Suss, J., & Ward, P. (2012). Use of an Option Generation Paradigm to Investigate Situation Assessment and Response Selection in Law Enforcement. *Proceedings of the Human Factors and Ergonomics Society Annual Meeting*, *56*(1), 297-301. https://doi.org/10.1177/1071181312561069

Suss, J., & Ward, P. (2018). Revealing perceptual–cognitive expertise in law enforcement: an iterative approach using verbal-report, temporal-occlusion, and option-generation methods. *Cognition, Technology & Work*, *20*(4), 585-596. https://doi.org/10.1007/s10111-018-0493-z

Ward, P., Suss, J., Eccles, D. W., Williams, A. M., & Harris, K. R. (2011). Skill-based differences in option generation in a complex task: a verbal protocol analysis. *Cognitive Processing*, *12*(3), 289-300. https://doi.org/10.1007/s10339-011-0397-9

Wigsten, A. (2020). *Polisiärt beslutsfattande i kritiska situationer*. Södertörns högskola (Södertörn University).
